# Multifactorial Nature of Fatigue in North Indian Rheumatoid Arthritis Patients

**DOI:** 10.31138/mjr.150124.mnf

**Published:** 2024-01-15

**Authors:** Alka Yadav, Chanchal Gera, Gurcharan Lal Avasthi

**Affiliations:** 1 Department of Internal Medicine, SPS Hospital, Ludhiana, India,; 2 Department of Internal Medicine and Rheumatology, SPS Hospital, Ludhiana, India

**Keywords:** rheumatoid arthritis, fatigue, vitamin D, disease activity, pain, sleep quality

## Abstract

**Introduction:**

Fatigue is a common, disabling, and poorly understood aspect of Rheumatoid arthritis (RA) treatment. Better understanding of fatigue is required for holistic treatment of RA. The present study was conducted to evaluate factors (disease activity, pain, sleep quality, and vitamin D) contributing to fatigue in RA

**Method:**

A cross-sectional study was conducted on 204 patients of RA. Fatigue was measured using CFQ-11 scale, pain and sleep impairment were assessed on visual analogue scale, disease activity by DAS 28 ESR, and vitamin D levels by enzyme chemiluminescence immunoassay. Univariate and multivariate binary logistic regression analyses were done to study association.

**Results:**

Mean age of study subjects was 51±11.63 years with majority (89.7%) being females and mean duration of RA was 8.54 years. Prevalence of fatigue was 66.2% (CFQ-11 score >4/11). Deficiency of vitamin D was found in 12.3% subjects. Mean sleep impairment and pain score on VAS were 32.60±21.53 and 26.37±21.65 respectively. Univariate analysis revealed that CFQ-11 fatigue score was independently associated with disease activity, pain, sleep, and vitamin D deficiency. Further Multivariate binary logistic regression analysis showed strongest association of vitamin D deficiency with fatigue (OR of 6.38 with 95% confidence interval of 1.58, 25.71). Disease activity (OR – 1.714, 95% CI- 1.14, 2.55) and sleep impairment (OR – 1.038, 95% CI- 1.005, 1.071) have also been found to be significantly associated with fatigue.

**Conclusion:**

Fatigue in RA is multifactorial, and it is mediated by disease-related factors (disease activity, sleep impairment) and non-disease-related factors (vitamin D deficiency).

## PRACTITIONER POINTS:

1. Fatigue in Rheumatoid Arthritis is common and disabling.
2. Despite its perceived importance and increased research in recent times, our lack of understanding of factors responsible for fatigue leads to poor outcomes and hence it is poorly managed.
3. Present study explores multiple factors contributing to fatigue in Rheumatoid Arthritis.

## BACKGROUND

The prevalence of Rheumatoid Arthritis (RA) in western countries is 0.5–1.0% and 0.7% in the adult Indian population.^1^ RA is an autoimmune inflammatory disorder that causes synovial inflammation and damage. RA as a systemic disease may also lead to a variety of extraarticular manifestations including fatigue, subcutaneous nodules, lung involvement, pleuritis, peripheral neuropathy, vasculitis, and haematological abnormalities.^2^ Majority of the patients present with pain, disability, and fatigue.^3^ Among all the manifestations of RA, fatigue is now considered as a common and disabling problem.^4^ Approximately 80–93% patients with RA experience fatigue^5^ and up to 74% patients experience persistently high and worsening level of fatigue.^3^ Fatigue in RA can be due to disease related aspects including pain, disease activity, and disease unrelated aspects including sleep, health-related quality of life, disability, work-related stress, and social support. In non-rheumatological conditions, additional factors such as hypothyroidism, anaemia, and vitamin D deficiency have been found to be linked to fatigue.^6^

Inflammation is proposed to cause fatigue both directly and indirectly. Fatigue is attributed to elevated levels of several inflammatory cytokines, ie, TNF α (Tumor Necrosis Factor) and IL-6 (interleukin 6). These cytokines cause dysregulations of the hypothalamic-pituitary-adrenal axis, thyroid insufficiency, and excessive formation of free radicals.^7^ A meta-analysis of therapeutic studies in RA have shown that blocking the action of these proinflammatory cytokines by biologics, ie, Anti TNF α, anti-IL6, and Anti-CD20 led to significant reduction in level of fatigue. Inflammation indirectly leads to fatigue by causing alterations in sleep wake cycle.^8^

A large proportion ie 40–75% of individuals with RA report sleep disturbances including poor quality of sleep, nighttime awakenings, and nonrestorative sleep.^9^ Disturbed sleep leads to excessive day time sleepiness and tiredness. Ranjbaran et al. described a vicious cycle of sleep impairment causing low pain thresholds which in turn leads to sleep disturbance.^9^

Vitamin D has a physiological role in maintenance of innate and acquired immunity as vitamin D receptors are present in primary lymphoid organs.^10^ It has modulatory effect on cells of immune system and decreases levels of proinflammatory cytokines.^11^ In vitro studies have shown vitamin D deficiency causes inflammation by modulating action of helper T cells and regulatory T cells.^12^ Vitamin D supplementation is found to have significant improvement in fatigue in otherwise healthy persons with vitamin D deficiency and also in patients with established RA.^13^

Fatigue has a significant contribution to the impaired quality of life, thus increasing the disease burden. Despite its perceived importance and increased research in recent times, our lack of understanding of factors responsible for fatigue leads to poor outcome; hence, fatigue is inappropriately managed. So, the objective of this study is to get more insight about the disease related and unrelated factors contributing to fatigue in RA population.

## METHODS

### Patients

A total of 204 classified patients of RA as per ACR/EULAR 2010 classification criteria attending a tertiary care hospital in North India were consecutively selected and enrolled over 1 year from April 2021 to April 2022.

### Inclusion and exclusion criteria

All the subjects classified as RA, above 18 years of age, and with consent to participate were included. Subjects of other rheumatological disorders, malignancy, chronic fatigue syndrome were excluded from the study.

### Ethical approval

Study was approved by institutional ethical committee (Registration number- ECR/155/Inst/PB/2013/RR-19) vide acceptance letter number SPS-I/2021/04 dated 22.03.2021.

### Assessment

Written informed consent was taken from all the participants. A thorough history, general physical examination, systemic examination, and lab investigations including ESR and vitamin D levels were done.

Fatigue was assessed by using Chalder Fatigue Questionnaire (CFQ 11). CFQ-11 score was used in 2 steps, first to find out whether fatigue is present or not, using Binary scoring system. In binary scoring system “better than usual’ and “no worse than usual” were scored as 0 while “worse than usual” along with “much worse than usual” were scored as one. Total score on binary scoring ranged from 0–11 and a score of ≥4 was considered equivalent to fatigue.

In second step, ordinal scale was applied to fatigue group to find out its severity. Each of the 11 items were answered on a 4-point ordinal scale ranging from the asymptomatic to maximum symptoms, such as “Better than usual” (0), “No worse than usual” (1), “Worse than usual” (2) and “Much worse than usual” (3). Point 1–7 measure physical fatigue and 8–11 measure psychological fatigue (annexure I). Total score on ordinal scale ranged from 0–33 on ordinal scale.

Disease activity was assessed by using DAS 28-ESR criteria (Disease Activity Score 28- Erythrocytic Sedimentation Rate) (Annexure II) which included number of swollen and tender joints, ESR, and global health assessment. Disease activity was classified into 4 groups:

1. High disease activity (DAS 28-ESR score- >5.1),
2. Moderate disease activity (DAS 28-ESR score 3.2 – 5.1),
3. Mild disease activity (DAS 28-ESR score 2.6 – 3.2),
4. Remission (DAS 28-ESR score <2.6).

Vitamin D status was assessed by measuring serum levels of 25(OH)vitamin D by enzyme chemiluminescence immunoassay and a level of <20 ng/ml was considered deficient and levels from 20–30 ng/ml were considered insufficient.

Pain was evaluated on a 100 mm Visual Analogue Scale (VAS) in which 0 being no pain and 100mm being worst pain (annexure III).

Sleep was evaluated using VAS with 0 being no sleep impairment and 100 being not able to sleep (annexure IV).

### Statistical analysis

Data were described in terms of range; mean ±standard deviation (± SD), frequencies (number of cases), and relative frequencies (percentages) as appropriate. To determine whether the data were normally distributed, a Kolmogorov-Smirnov test was used. Comparison of quantitative variables between the study groups was done using Student t-test and Mann Whitney U test for independent samples for parametric and non-parametric data respectively. For comparing categorical data, Chi square (χ2) test was performed, and Fisher exact test was used when the expected frequency is less than 5. Covariates obtaining a probability value (p value) <0.05 in the univariate analyses were included in the multivariate Binary logistic regression analyses. A p value less than 0.05 was considered statistically significant. All statistical calculations were done using (Statistical Package for the Social Science) SPSS 21version (SPSS Inc., Chicago, IL, USA) statistical program for Microsoft Windows.

## RESULTS

### Patient characteristics

A total of 204 RA patients were enrolled from April 2021 to April 2022 and the participant characteristics are described in **Table 1**. Mean age of study subjects was 51 years (SD, 11.63) ranging from 19 years to 74 years old. There were 183 (89.7%) females and 21 (10.3%) males with Female to Male ratio of 8.7:1. Mean duration of illness of study subjects was 8.54 years (SD, 8.39). Most common presenting problems were pain, fatigue, and joint swelling in 82.4%, 65.2%, 40.2% subjects respectively. Majority (84.8%) of the patients participating in the study were on DMARDs (Disease-Modifying Anti Rheumatic Drugs), rest of the subjects were either newly diagnosed or were taking homeopathic/ayurvedic medication. Methotrexate was most commonly used DMARD. On the basis of CFQ 11 bimodal score, study participants were divided into 2 groups (fatigued and non-fatigued). In our study, prevalence of fatigue was 66.2%, ie, CFQ-11 score >4 out of 11. The remaining 33.8% subjects were classified as non-fatigued. Mean CFQ 11 score was 5.13 (SD, 3.28). Disease activity assessment using DAS28-ESR showed mean disease activity score of study subjects was 3.62 (SD, 1.65), mean pain score was 32.60 (SD, 21.53) and mean sleep impairment was 26.37 (SD, 21.65) on a scale ranging from 0–100. Mean vitamin D level of study subjects was 43.97 (SD, 19.86). Majority subject (76%) had normal levels of vitamin D, 12.3% subjects had vitamin D deficiency and 11.8% had insufficient levels.

**Table 1.** Characteristics of study participants (N=204).

**Variable**	**N (%)**
**Age, years**	51
**Gender**	
Females	183 (89.7%)
Males	21 (10.3%)
**Disease duration, years**	8.54
**Presenting complaints**	
Pain	168 (82.4%)
Swelling	82 (40.2%)
Disability	15 (7.4%)
Fatigue	133 (65.2%)
Sleep impairment	16 (7.8%)
**Medications**	
DMARDs	173 (84.8%)
Iron	16 (7.9%)
Folic acid	82 (40.2%)
Thyroxine	21 (10.3%)
Vitamin D	132 (64.7%)
Antidepressants	22 (10.8%)
**Comorbidities**	
T2DM	17 (8.3%)
HTN	38 (18.6%)
Hypothyroidism	22 (10.8%)
Heart disease	6 (2.9%)

### Univariate analysis

Each variable was assessed for its degree of association with fatigue. Variables with p value <0.05 were considered statistically significant. t test was used for DAS28 and Mann Whitney U test was used for rest of the parameters. Univariate analysis showed that CFQ-11 score was significantly associate with disease activity, pain, sleep, and vitamin D deficiency (**Table 2**).

**Table 2.** Univariate analysis of factors contributing to fatigue in rheumatoid arthritis.

**Variable**	Non-Fatigue group CFQ-11* score < 4 (N=71)		Fatigue group CFQ-11* score > 4 (N=133)		**T**	**p-value**
	**Mean**	**SD**	**Mean**	**SD**		
DAS 28 Score	2.65	1.22	4.12	1.62	−6.625	0.001
Pain score (VAS)	18.99	16.73	39.56	20.40	−7.223	0.001
Sleep Impairment (VAS)	12.61	12.68	33.41	21.93	−7.276	0.001
Vitamin D (ng/ml)	49.01	17.18	41.39	20.68	2.632	0.004

* Chalder fatigue scale SD: Standard deviation.

### Multivariate analysis

Multivariate binary logistic regression analysis was done on the covariates which were significantly associated with fatigue on univariate analysis (**Table 3**). Vitamin D deficiency had strongest positive association with fatigue. Vitamin D level <20 mg/dl is more strongly associated with fatigue with an odd’s ratio of 6.38 (95% CI-1.58, 25.71), Whereas levels between 20–30mg/dl had an odd’s ratio of 4.18 (CI- 1.18, 14.83). Disease activity and sleep impairment also remained significantly associated with fatigue with odds ratio of 1.71 (95% CI- 1.14, 2.55) and 1.03 (95% CI-1.00, 1.07) respectively.

**Table 3.** Multivariate regression analysis of factors contributing to fatigue in rheumatoid arthritis (N=133).

**Variable**	**p-value**	**Odds ratio**	**95% C.I. for Odds ratio**	
			**Lower**	**Upper**
DAS28 Score	0.008	1.714	1.148	2.559
Pain Score (VAS)	0.367	1.013	0.985	1.041
Sleep Impairment (VAS)	0.022	1.038	1.005	1.071
Vitamin D (ng/ml) > 30	0.005			
20–30	0.027	4.184	1.180	14.839
<20	0.009	6.385	1.585	25.715

## DISCUSSION

Fatigue is one of the common symptoms in the variety of rheumatological disorders as well as in the general population.^14^ It is difficult to interpret cause of fatigue in clinical practice. Various studies have been held in the past to explore the multidimensional nature of fatigue in RA. Our study is an attempt to add to our knowledge about the prevalence and correlation of fatigue with various factors in patients with RA. This study was conducted in a Tertiary care hospital in North India. In this cross- sectional study, the prevalence of fatigue was 66.2%. Studies done by Hoogmoed et al.^3^ and Repping-Wuts et al.^15^ found similar prevalence of severe fatigue in 42% and 50% of the patients respectively. Both these studies used CIS (Checklist Individual Strength) to assess fatigue. Very high prevalence (80%) has also been reported.^16^ Wide variation in prevalence of fatigue as described in above mentioned studies can be attributed to the different assessment methods used and different threshold of fatigue with each scale. The dimension of fatigue measured also differs with each scale.

Though the majority (76%) of our subjects had sufficient vitamin D level because most of them were already receiving Vitamin D, in our study, we observed strongest association of fatigue with vitamin D deficiency. Odds of fatigue was 6.3 with vitamin D deficiency and 4.1 with insufficient levels, indicating a linear relationship. Studies in the past have shown a significant prevalence of vitamin D deficiency among otherwise healthy individuals presenting with fatigue and improvement in fatigue after vitamin D supplementation,^13^ but literature for RA patients is limited. In contrast to our results, a study conducted by Jelsness-Jørgensen et al.^11^ on 169 patients of established RA did not find any significance of deficient vitamin D on fatigue.

The association between fatigue and DAS-28 was statistically significant in our study in both the univariate as well as the multivariate analysis. Studies done in past have shown conflicting results for disease activity as a contributing factor for fatigue. A strong association between disease activity and fatigue was observed in a cross-sectional study conducted by Singh et al.^17^ Pollard et al.^16^ used VAS to assess fatigue which correlated with disease activity on simple regression, but not on multiple regression, indicating the association of disease activity with fatigue is secondary. The reason for conflicting results may be because of different comorbidities in our population and different fatigue measurement tool used by us. Pain is amongst the most common presenting complaints in RA patients. Present study observed association of pain with fatigue on univariate analysis but not on further multivariate regression analysis. There is literature supporting pain as one of the major contributing factors of fatigue in a cross-sectional study conducted by Pollard et al.,^16^ in which pain had strongest association with fatigue on multiple linear regression analysis. Another study by Hoogmoed et al.^3^ evaluated physical and psychological correlates of fatigue on 228 patients of RA showed an association between pain and fatigue on univariate analysis. Huyser et al.,^18^ Thyberg et al.^4^ and Campbell et al.^19^ also corroborated similar findings in their respective studies. Another study found no association of fatigue with pain score.^14^ Thus, the contribution of pain in fatigue is questionable. Although fatigue in Rheumatoid Arthritis has been studied in the past, literature is sparce on its multidimensional nature. The present study was aimed to evaluate multiple disease-related and unrelated factors contributing to fatigue and their association with fatigue.

Greater insight into the aetiology aids in better management of patient symptoms. Limited research has been done in past on the population chosen (ie, north Indian population) in present study.

## LIMITATIONS

This study possessed some limitations. Direct causal relationship of these factors could not be ascertained given the cross-sectional study design. We have not assessed pattern of change in fatigue. Prospective longitudinal studies are required for studying causation and improvement in fatigue with therapeutic interventions. Further consistency of data can be validated by doing multi-centre study and by sampling at multiple time points.

## CONCLUSION

The study has shown that fatigue is one of the troublesome problems faced by patients with rheumatoid arthritis with a prevalence of 66.2%. Aetiology of fatigue is multifactorial, and patients complains of fatigue despite control of disease activity and pain. In the present study, vitamin D deficiency, disease activity, and sleep impairment were found to be independently associated with fatigue. Treatment of fatigue needs other methods beyond treatment of disease activity.

## APPENDICES

### Appendix I.

#### Chalder Fatigue Questionnaire-11 (CFQ-11).

**Table:**

**Question**	**0**	**1**	**2**	**3**
1.Do you have problem with tiredness?				
2.Do you need to rest more?				
3.Do you feel more sleepy or drowsy?				
4.Do you have problems starting things?				
5.Do you lack energy?				
6.Do you have less strength in your muscles?				
7.Do you feel weak?				
8. Do you have difficulty concentrating?				
9.Do you make slips of the tongue when speaking?				
10.Do you find it more difficult to find the right word?				
	**3**	**2**	**1**	**0**
11. How is your memory?				

0: better than usual; 1: no worse than usual; 2: worse than usual; 3: much worse than usual.

### Appendix II.

Disease activity score 28.

DAS28(ESR) = 0.56*√(TJC28)+0.28*√(SJC28)+0.014*GH+0.70*ln(ESR)

### Appendix III.

Visual analogue scale for pain assessment.

### Appendix IV.

Visual analogue scale for sleep impairment assessment.
